# Influence of Health Behaviors and Occupational Stress on Prediabetic State among Male Office Workers

**DOI:** 10.3390/ijerph15061264

**Published:** 2018-06-14

**Authors:** Hosihn Ryu, Jihyeon Moon, Jiyeon Jung

**Affiliations:** College of Nursing, Korea University, Seoul 02841, Korea; hosihn@korea.ac.kr (H.R.); hepburn86@korea.ac.kr (J.J.)

**Keywords:** prediabetic state, health behavior, occupational stress, male, office worker

## Abstract

This study examined the influence of health behaviors and occupational stress on the prediabetic state of male office workers, and identified related risks and influencing factors. The study used a cross-sectional design and performed an integrative analysis on data from regular health checkups, health questionnaires, and a health behavior-related survey of employees of a company, using Spearman’s correlation coefficients and multiple logistic regression analysis. The results showed significant relationships of prediabetic state with health behaviors and occupational stress. Among health behaviors, a diet without vegetables and fruits (Odds Ratio (OR) = 3.74, 95% Confidence Interval (CI) = 1.93–7.66) was associated with a high risk of prediabetic state. In the subscales on occupational stress, organizational system in the 4th quartile (OR = 4.83, 95% CI = 2.40–9.70) was significantly associated with an increased likelihood of prediabetic state. To identify influencing factors of prediabetic state, the multiple logistic regression was performed using regression models. The results showed that dietary habits (β = 1.20, *p =* 0.002), total occupational stress score (β = 1.33, *p =* 0.024), and organizational system (β = 1.13, *p =* 0.009) were significant influencing factors. The present findings indicate that active interventions are needed at workplace for the systematic and comprehensive management of health behaviors and occupational stress that influence prediabetic state of office workers.

## 1. Introduction

The economically active population in Korea has been reported to be 62.3% of about 43 million people aged 15 years or older [1]. According to the 2018 data [1] of the National Statistical Office, male workers constitute 57.7% of the economically active population, and office workers such as managers and professionals, were reported to comprise 60.9% of all occupational clusters. Since office workers perpetually perform document processing, communication, information management, and decision-making tasks mostly in offices [2], not only personal factors but also workplace or environment characteristics affect worker health, as they spend most of their daily time in the workplace [3,4]. Such workplace-related factors also affect the level of personal health and unhealthy health behaviors [3,4]. In particular, a previous study which investigated the health behaviors of office workers in Korea reported that 78.4% of the participants consumed alcohol more than 1–2 times per week [5], and a study [6] that surveyed 4457 randomly sampled office workers reported that 47.8% of the male workers were smokers. Compared to workers in other types of occupations, office workers have insufficient levels of physical activity due to the sedentary nature of their work [3,4,7], and their average energy intake per 1 kg of body weight per day has been reported to be higher than the standard value for their level of activity, along with a high intake of fat [8,9]. In addition, occupational stress among office workers arising from work-related factors, such as job strain, relationships with superiors and colleagues in the workplace, and anxiety about their positions (e.g., early retirement), have been reported to be relatively higher than that among blue-collar workers [7].

Factors related to occupational stress and health behaviors lead to health problems among office workers, and the prevalence of chronic diseases, such as diabetes, obesity, hypertension, and hyperlipidemia have been reported to be increasing steadily [3,4], according to the data analysis of a national health screening program conducted among workers. Furthermore, occupational stress affects the prevalence of cardiovascular diseases and causes problems with normal metabolism, and in particular, it has been reported to be a major cause of diabetes [10]. Previous studies on workers [10,11] have also reported that the concept of occupational stress, which includes job demands, job strain, and effort–reward imbalance, is related to the prevalence of diabetes among male workers. 

From 2012 to 2016, the prevalence of prediabetic state and diabetes among adults aged 30 years or older in Korea consistently increased from 19.9% to 24.8%, and from 10.1% to 13.7%, respectively, and the prevalence of diabetic complications was also high [12]. In addition, a 10-year cohort analysis of office workers in large corporations [3] showed health risks of triglyceride and increases in waist circumference as overtime for such workers, and the risk of contracting diabetes was 3.7 times, which was the highest.

According to the criteria of the American Diabetes Association (ADA), the fasting blood sugar level for prediabetic state is between 100 mg/dL and 125 mg/dL [13], and about 5–10% of prediabetic people develop diabetes [12,14,15,16]. Prediabetic state not only increases insulin resistance and exacerbates β-cell function, but also affects organs such as the eyes, kidneys, blood vessels, and heart, leading to neuropathy, diabetic retinopathy, and cardiovascular diseases [17]. Progression to diabetes can be prevented, and fasting blood sugar can be returned to the normal range (under 100 mg/dL) by changing health behaviors related to weight control, dietary habits, and physical activity [17]. However, because prediabetic people do not feel the need to control their blood sugar level and change their health behaviors due to their relatively lower knowledge and interest in their own blood sugar level compared to those diagnosed with diabetes, they do not engage in proper health management [15]. 

Many studies have reported that prediabetic state is related to health behaviors such as smoking [18,19], drinking [20,21], physical activity [22], and dietary habits [23]. Moreover, occupational stress, including job demands and effort–reward imbalance, is also related to the prevalence of diabetes [10,11]. Research that has comprehensively analyzed the relationship among occupational stress, health behaviors, and prediabetic state among office workers, and identified that influencing factors of prediabetic state is nonetheless lacking. Systematic health management of office workers who are exposed to unhealthy health behaviors is needed [3]. In particular, if preventive intervention programs are applied based on the knowledge that the development of a prediabetic state into diabetes can be prevented through proper prevention and early interventions [17], the effect of such health management is expected to be quite large. Accordingly, the present study was conducted on male office workers of one company to analyze the effect of health behaviors and occupational stress on prediabetic state.

## 2. Materials and Methods 

### 2.1. Methods

The present study is a cross-sectional study conducted on male office workers of a single company located in Seoul, Korea. The subjects of the present study were selected from all the workers currently employed (about 1300 workers in 2017) by the company according to the following procedures. 

To collect data on 2014 regular health checkups, and the results of a health screening questionnaire for all the workers, the purpose and efficiency of the study were explained to the health manager of the company, and permission was obtained to acquire the data. Random number IDs were used for all data on regular health checkups and health screening questionnaires before the data were collected, so that no individual could be identified. A health behavior survey was carried out through the company intranet for a total of three months. Only the participants who agreed to the use of the survey data were allowed to respond to the survey, and 764 people (58.7%) of all 1300 employees completed the survey. Among the respondents who completed the survey, 550 (72.0%) were male. Participants who had been diagnosed with diabetes (19 participants whose fasting blood sugar exceeded 125 mg/dL) and 256 participants whose responses were incomplete (74 workers who were in a prediabetic state, 181 workers whose fasting blood sugar was within the normal limits) were excluded. Consequently, a total of 275 participants were included in the analysis of the present study to compare individuals with a normal fasting blood sugar level with individuals in a prediabetic state. 

To protect the rights and interests of the participants, the present study was conducted after obtaining approval (KU-IRB-13-12) from the Institutional Review Board.

### 2.2. Measures

Regular health checkups included blood tests, anthropometric measurements, and blood pressure measurements. Fasting blood sugar was measured by a blood test. Following the criteria of the ADA [13], people whose fasting blood sugar level was between 100 mg/dL and 125 mg/dL were included in the prediabetic state group, and those whose fasting blood sugar level was less than 100 mg/dL were included in the normal group. Anthropometric measurements included body weight, height, body mass index (BMI), and waist circumference. Body weight and height were used to calculate BMI, which was obtained by dividing body weight (kg) by height (m^2^). Blood pressure included diastolic blood pressure and systolic blood pressure, and it was measured by health care provider at the time of regular health checkups. Regular health checkups were conducted at a designated health checkup hospital based on the Korean Occupational Safety and Health Act, and all measurements were made according to standardized manuals.

The health questionnaire conducted at the time of the regular health checkups included questions regarding smoking, drinking, and physical activities. Smoking, drinking, and physical activities correspond to four health behaviors (smoking, drinking, physical activities, and dietary habits) related to noninfectious diseases announced by the World Health Organization (WHO) [24]. Smoking was classified according to past and present smoking status (never smoked, smoked before but quit, currently smoking). Drinking is a quantitative concept measured by average daily drinking during the past one year. It was measured by average daily drinking (not drinking or less than one drink, one drink to less than five drinks, five drinks or more) in the light of the criteria of the National Institute of Alcohol Abuse and Alcoholism (NIAAA) [25]. In addition, physical activities were measured by the frequency of exercise intense enough to become damp with sweat per week (5–6 times (almost every day), 3–4 times, 1–2 times, no exercise at all).

The health behavior survey included dietary habits among health behaviors and occupational stress. Dietary habits captured the intake of various seasonal fruits five times or more per week and two or more types of vegetables in various colors per meal during the past week (had both fruits and vegetables, had fruits or vegetables, did not have fruits or vegetables). For occupational stress, the Korean Occupational Stress Scale-Short Form (KOSS-SF) was used, which was developed by the Korea Occupational Safety and Health Agency [26]. KOSS-SF is developed and standardized to measure unique and specific occupational stressors in Korean workers, and used by most domestic businesses [26]. The instrument comprises a total of 24 items in seven subareas, including four items on job demands, four items on lack of job autonomy, three items on interpersonal conflict, two items on job insecurity, four items on the organizational system, three items on lack of reward, and four items on the occupational climate. Each item is measured on a Likert scale (one point for strongly disagree, two points for disagree, three points for agree, and four points for strongly agree), where higher total scores indicate higher occupational stress. The reliability of the instrument at the time of development measured by Cronbach’s α was 0.51–0.85 [26], and Cronbach’s α in the present study was 0.82, with α coefficients for the subscales ranging from 0.81 to 0.83. In the present study, the total score and converted score of each domain were converted into a full score of 100 points, and scores were then classified into quartiles based on the evaluation reference values of the KOSS-SF. Analysis was performed on the 1–2 quartile (which was formed by combining the 1st and 2nd quartiles), 3rd quartile, and 4th quartile, where the 1–2 quartile was used for reference [27]. The conversion equation was as follows:

- Converted score of each domain = {(actual score − number of items)/(highest predictable score − number of items)} × 100
- Total score of occupational stress = sum of converted scores of seven domains/7

### 2.3. Statistical Analysis

The Statistical Package for the Social Sciences (SPSS) statistics 23.0 program (IBM Corporation, New York, USA) was used to analyze the data, and the specific analysis method was as follows. Descriptive statistics were calculated to determine the sociodemographic characteristics, health-related characteristics, health behaviors, and occupational stress characteristics of male office workers. To identify the difference between health behaviors and occupational stress according to the existence of prediabetic state, a *t*-test and χ^2^ test were performed, and the correlations between prediabetic state and the measured variables were analyzed using Spearman’s correlation coefficients. In addition, multiple logistic regression analysis was used to analyze the risk of health behaviors and occupational stress for prediabetic state. To identify the influencing factors of prediabetic state, stepwise multiple logistic regression was performed using regression models. Among the statistically significant variables in the descriptive statistical analysis and risk analysis, working years, marital status, waist circumference, systolic blood pressure, and diastolic blood pressure were included to perform multiple logistic regression, but age, body weight, and BMI were excluded, since working years were highly correlated with age and waist circumference were highly related with body weight and BMI, and working years and waist circumference were important factors for office workers [3,28].

## 3. Results

### 3.1. Sociodemographic and Health-Related Characteristics

The results of the comparison of sociodemographic and health-related characteristics between the normal group and prediabetic group (Table 1) showed that the average working years for the normal group and prediabetic group were 7.64 and 10.60 years, respectively, which indicates that individuals in the prediabetic group have worked longer than those in the normal group (*p =* 0.003). In addition, the average waist circumference of the prediabetic state group was 87.38 cm, which was greater than the average waist circumference of 84.53 cm for the normal group (*p =* 0.006). 

### 3.2. Status of Health Behaviors and Occupational Stress

In terms of drinking among health behaviors, about 11% among the participants drank “five drinks or more” in the prediabetic group, which was about five times greater in terms of percentage than the four participants (2.2%) who did so in the normal group. In the case of physical activities, the distribution of “5–6 times (almost every day)” was found to be about five times higher in the normal group than in the prediabetic state group (*p =* 0.011). For occupational stress, the total score of 44.67 for the prediabetic state group was higher than the score of 39.11 for the normal group (*p <* 0.001). The proportion in the 4th quartile, which signifies high occupational stress, for the prediabetic state group was higher than that for the normal group, while the proportion in the 1–2 quartile, which signifies low occupational stress, was lower for the prediabetic state group than for the normal group. 

### 3.3. Correlation of Health Behaviors and Occupational Stress

In the case of health behaviors, the prediabetic state group showed stronger correlations with all subfactors of smoking (*r =* 0.37, *p <* 0.001), drinking (*r =* 0.53, *p <* 0.001), physical activities (*r =* 0.34, *p =* 0.001), and dietary habits (*r* = 0.58, *p <* 0.001) than the normal group. In terms of occupational stress, the prediabetic state group showed stronger correlations with the total score and scores of all subscales than the normal group (Table 2). 

### 3.4. Odds ratio of Health Behaviors and Occupational Stress 

**Figure 1:**
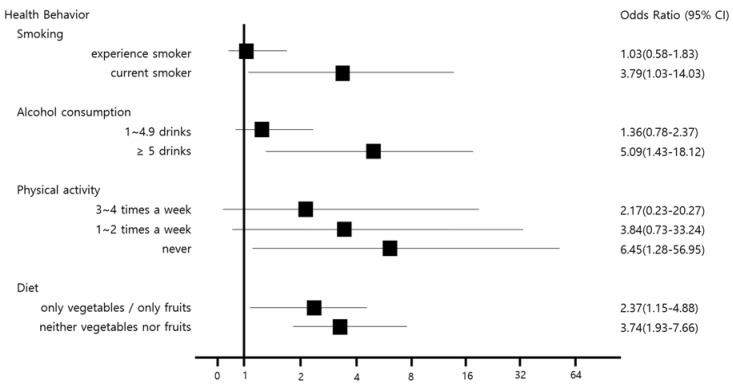
Odds ratio (OR) of health behaviors including smoking, alcohol consumption, physical activity, and diet. The OR was marked to the square points, and adjusted for working years, marital status, waist circumference, systolic blood pressure, and diastolic blood pressure. References were “Never” on smoking, “None or <1 drink” on alcohol consumption, “5~6 times a week” on physical activity, and “Both vegetables and fruits” on diet. CI = confidence interval.

**Figure 2:**
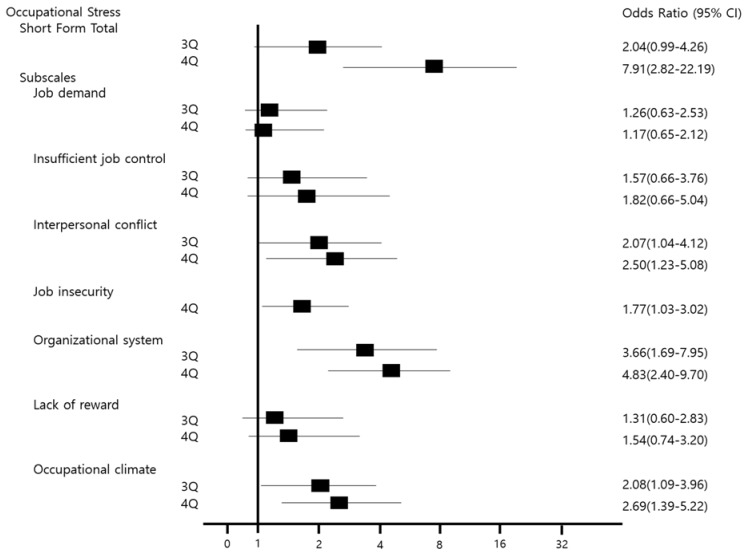
Odds ratio (OR) of occupational stress. The OR was marked to the square points and adjusted for working years, marital status, waist circumference, systolic blood pressure, and diastolic blood pressure. References were 1–2 Quartile (1–2 Q). CI = confidence interval; 3 Q = 3 Quartile; 4 Q = 4 Quartile.

The results of the analysis of the risk of health behaviors and occupational stress for prediabetic state are shown in Figure 1 and Figure 2. For occupational stress, the risk in the 4th quartile was higher for the total occupational stress score and the scores of each subscale than the risk in the 1–2 quartile. The risk of interpersonal conflict, job insecurity, organizational system, and occupational climate among the subscales was statistically significant. 

### 3.5. Factors Influencing Prediabetic State 

Table 3 shows the results regarding the influencing factors of office workers’ health behaviors and occupational stress for prediabetic state. For Model 1, regression analysis was performed by including only the significant variables in the risk analysis for each explanatory variable to consider the effect on other explanatory variables. For Model 2, analysis was performed by adding working years, marital status, waist circumference, and diastolic and systolic blood pressure. Both models were statistically significant (Model 1: χ^2^ = 45.96, *p <* 0.001, Model 2: χ^2^ = 58.87, *p <* 0.001), and the explanatory power of Models 1 and 2 according to the Nagelkerke coefficient of determination were 21.4% and 26.7%, respectively. The classification accuracy of Models 1 and 2 was 74.5% and 73.8%, respectively, and the goodness of fit of the models performed using the Hosmer–Lemeshow test failed to reject the hypothesis that there is no difference between the observed value and predicted value of the model, (Model 1: χ^2^ = 5.11, *p =* 0.746, Model 2: χ^2^ = 11.30, *p =* 0.185), which indicates that the presented models coincide with the data well. 

In Model 1, drinking and dietary habits among health behaviors and job insecurity and organizational system among the subscales of occupational stress were found to be significant influencing factors of prediabetic state. In Model 2, dietary habits, total occupational stress score, organizational system, working years, and waist circumference were found to be significant influencing factors of prediabetic state. In particular, total occupational stress score and organizational system within the 4th quartile and “did not have fruits or vegetables” were found to act as significant influencing factors of prediabetic state.

## 4. Discussion

Of the 550 male office workers who completed the survey, 166 workers were in a prediabetic state, which indicates a prevalence of 30.4%. This figure is higher than the average prevalence of 24.8% [12] among Korean adults aged 30 years or older. In addition, the prevalence of prediabetic state in the present study is higher than that of previous studies that did not consider gender and occupation, since the prevalence of prediabetic state is generally higher among males than among females and in office jobs than in manufacturing jobs [29]. 

Many previous studies [14,30,31,32] have considered BMI, body weight, and physical activity as the main influencing factors in workplace-based diabetes prevention programs. However, the results of the present study showed that dietary habits, total occupational stress score, organizational system, working years, and waist circumference are the main influencing factors of prediabetic state among workers. 

In particular, in the case of dietary habits, previous studies have confirmed that the intake of fruits and vegetables is an important factor that reduces the prevalence of diabetes [23,33]. Yin et al. [23] reported findings similar to that of the present study in that the risk of prediabetic state, and diabetes increases when fruits and vegetables are rarely consumed or not consumed at all, compared to when they are frequently consumed. The slight difference in the risk of prediabetic state between the present study and previous studies appears to be due to the differences in the recommended standards for dietary habits. Meanwhile, the results of a meta-analysis of type 2 diabetes mellitus showed that the intake of fruits or vegetables, especially green leafy vegetables, reduces the risk of diabetes, but no significant benefit of consuming fruits and vegetables together was found [34]. This divergence in the findings may be due to the use of a different instrument from that of the present study since the analysis was conducted after standardizing the intake of fruits or vegetables to daily intake (106 g). Therefore, not only the simple act of consuming fruits or vegetables but also the amount of intake per day needs to be considered in future studies.

Regarding the total occupational stress score, Djindjic et al. [10] reported a significant association between occupational stress and diabetes. Particularly, in the case of male workers, higher occupational stress was associated with a higher prevalence of diabetes, and it was found that the level of occupational stress was the highest among bank employees who have relatively longer working hours in a sitting position. A study that examined the association between work stress and diabetes and prediabetic state found that higher occupational stress is associated with diabetes and prediabetic state [35]. In addition, a closer look at the differences in occupational stress by diabetes status indicated that, particularly among men, workers with diabetes did not have the highest level of work stress; instead, the occupational stress of workers in the prediabetic state was the highest. Based on the findings of the present and previous studies, it is important that employees with diabetes, and those in prediabetic state, try to manage and reduce occupational stress as a preventive measure.

Regarding organizational system, which is a subscale of occupational stress, previous studies that have analyzed the relationships between organizational system and fasting blood sugar level or prediabetic state are lacking other than a study [36] carried out on Korean workers employed in various workplaces. A previous study conducted on 2097 workers who received health examinations at the health examination center of a university hospital reported that the multivariable-adjusted OR (95% CI) of the incidence of diabetes among those with job insecurity was 0.44 (95% CI = 0.25–0.76) in the “high” group (the group with above median value) group compared with the reference “low” (the group with below median value) group, but the influence of other subareas was nonsignificant [36]. This is a somewhat different finding from that of the present study, which found that organizational system is a major influencing factor of prediabetic state. This difference in the results may be attributed to the use of office workers as subjects in the present study, as the aforementioned study did not take social and occupational characteristics into consideration. Furthermore, the findings regarding the subareas of occupational stress that lead to significant increases in fasting blood sugar level were also somewhat different. Such differences may be due to the use of a different instrument from that used in the present study, which used the KOSS-SF, an occupational stress measurement instrument developed for Korean workers, as the previous study defined occupational stress using job-demand control (JDC) and effort-reward imbalance (ERI). 

According to a 10-year cohort analysis of office workers in large corporations [3], the OR of those who worked for over 20 years, compared to those who worked 1 to 2 years, were 2.70 (95% CI = 1.63–4.45) in waist circumference (male ≥90 cm), and the risk of contracting diabetes was 3.7 times, which was the highest. In addition, previous studies [28,37] reported that it was important to monitor and prevent increases in waist circumference to reduce the increasing burden of prediabetes, diabetes, and its complications. These are similar to the results of the present study and highlight the need for further studies to consider working years and maintaining waist circumference.

In addition to dietary habits, total occupational stress score, and organizational system that were identified as major influencing factors of prediabetic state, the analysis of the risk of health behaviors and occupational stress for prediabetic state showed that the risk of prediabetic state is high if the individual is currently smoking, drinks five or more drinks, and does not engage in physical activity, as well as if the scores on the subscales of occupational stress (i.e., interpersonal conflict, job insecurity, and occupational climate) are high. 

A previous study [19] that aimed to identify the relationship between smoking behavior among male workers and the prevalence of prediabetic state reported that the prevalence of prediabetic state is 1.12 times higher for those who have smoked 100 or more cigarettes during their lifetime, or those who are current smokers, than for nonsmokers, but the result was not statistically significant (*p =* 0.06). A study conducted on adults [18] reported that current smoking significantly increases the risk of prediabetic state (*p <* 0.001). This result is consistent with the results of the present study that the risk of prediabetic state for current smokers is higher than that for nonsmokers. Meanwhile, because the risk of prediabetic state increases when the amount of smoking is greater, and the duration of smoking cessation is shorter [38], it is necessary for future studies to consider not only smoking behavior in the past or present but also the amount of smoking and the duration of smoking cessation in the past or present.

Huang et al. [20] analyzed the risk of prediabetic state among current drinkers and nondrinkers, and determined that current drinking behavior increases the risk of prediabetic state. In addition, it has been reported that as the amount of drinking increases, the risk of diabetes increases up to 1.63 times, and the added risk for diabetes and prediabetic state increases up to 1.54 times [21,39], which suggest that moderate drinking is an important health behavior to prevent prediabetic state. 

Research on the direct association between physical activities and prediabetic state has been somewhat insufficient, but most studies have reported that moderate physical activity for 30 min or more every day reduces the risk of diabetes [22,40]. In particular, a previous study [22] conducted to identify the risk factors of diabetes among American males reported that the risk of diabetes decreases as the frequency of engaging in moderate physical activity for 30 min or more every day increases. That is, moderate physical activities for 30 min or more may have a positive effect on preventing prediabetic state and glycemic control. Meanwhile, Nakanishi, Takatorige, and Suzuki [41] reported that diabetes and prediabetic state progressively decreased by increasing energy expenditure on daily life activity, such as occupational physical activity, brisk walking, riding vehicles (standing position) to and from work. Therefore, it is necessary for future studies or interventions to consider not only moderate physical activity, but also daily life activities that are beneficial for managing fasting glucose.

Previous studies [10,11] conducted to determine the relationship among occupational stress, fasting blood sugar level, and risk of diabetes have reported the following results. Djindjic et al. [10] found that the high demand, strictness, and conflict/uncertainty are not associated with diabetes. However, a study that investigated the possible influence of work stress on diabetes reported that in men, high work demands and high job strain decrease the risk for diabetes, as did an active job (high demands and high decision latitude) [42]. These findings are somewhat different from the results of the present study, which found that subjects with high scores on the subscales of occupational stress (interpersonal conflict, job insecurity, and occupational climate) had a high risk for prediabetic state. That is, there is conflicting evidence regarding the subscales of occupational stress; therefore, it is necessary to carry out repeated research to determine whether its subscales are associated with diabetes or prediabetic state, and how they are related. 

Since the present study performed an integrative analysis on data from regular health examinations, health questionnaires, and a health behavior-related survey of all the employees of a company, it can be used as the results of a basic analysis for the development of a prediabetic intervention program. In particular, the significance of the present study lies in the determination of risks and correlations among health behaviors, occupational stress, and prediabetic state, as well as a systematic analysis of the influencing factors among male office workers who have been reported to have difficulties in practicing health behaviors and have experienced a high level of occupational stress. Nevertheless, the present study has the following limitations. First, since the study was conducted on male office workers working at a company at one point in time, the ability to identify cause-and-effect relationships accurately, or generalize the results, is somewhat limited. However, it would help to examine the associations among occupational stress, health behaviors, and prediabetic state of office workers and the influencing factors of prediabetic state. Second, the study sample was relatively small, and the response rate was low, since participation in the survey was voluntary. However, the results are meaningful because this study was conducted on all employees of a company. Finally, since the working environment and health characteristics of office workers could differ, repeated and further studies are needed to overcome such limitations, and in particular, repeated research through analysis of government-based datasets is needed. 

## 5. Conclusions

The prevalence of prediabetic state among male office workers who were the subjects of the present study was 30.4%, which was higher than the prevalence (24.8%) among general Korean adults aged 30 years or older. This finding implies the necessity of health promotion programs for high-risk groups for chronic disease, which is especially applicable to prediabetic state among the various health problems of office workers. Furthermore, based on the analysis of the present study, which showed a significant influence of dietary habits among health behaviors, total occupational stress score, and organizational system among the subscales of occupational stress on prediabetic state, organized changes of not only individual workers, but also workplaces, are needed for the systematic prevention and management of diabetes. In other words, comprehensive changes that include individual workers and the work environment need to be induced by preponderantly reflecting on matters related to dietary habits, total occupational stress score, and the organizational system in intervention programs targeting prediabetic state. The development of comprehensive intervention programs with considerations given to health behaviors and occupational stress-related variables for male office workers in a prediabetic state is also necessary, and large-scale expanded research is needed to identify the influencing factors of prediabetic state among Korean office workers. Finally, active and systematic interventions by the government are necessary for people in a prediabetic state who lack knowledge on health management compared with people who already have a chronic disease. The present study is the first study to collectively analyze health behaviors, occupational stress, and prediabetic state among office workers, and it is expected to be effectively utilized in workplace-based intervention programs conducted for office workers in the future.

## Figures and Tables

**Table 1:** Sociodemographic and Health-Related Characteristics in the Study Population.

Characteristics	Total (*N* = 275)	Normal (*N* = 183)	Prediabetic State (*N* = 92)	*t*/χ^2^	*p*-Value
	Mean ± SD or *n* (%)				
Sociodemographic Characteristics					
Age (years)	38.1 ± 8.15	36.8 ± 7.63	40.9 ± 8.49	−4.06	<0.001
Marital status				8.28	0.004
married	177(53.2)	107(58.5)	70(79.3)		
unmarried	98(35.8)	76(41.5)	22(20.7)		
Education level				3.59	0.058
University graduates	234(85.1)	161(88.0)	73(79.3)		
Graduate school	41(14.9)	22(12.0)	19(20.7)		
Working years (years)	8.63 ± 7.32	7.64 ± 6.80	10.60 ± 7.95	−3.05	0.003
Health-Related Characteristics					
FBS (mg/dL)	95.69 ± 11.00	89.99 ± 6.58	107.03 ± 9.06	−17.79	<0.001
Height (cm)	174.9 ± 5.29	174.7 ± 5.30	175.3 ± 5.30	−0.86	0.388
Body weight (kg)	75.5 ± 10.18	74.2 ± 10.08	78.0 ± 9.95	−2.93	0.004
BMI (kg/m^2^)	24.63 ± 2.84	24.27 ± 2.82	25.34 ± 2.74	−2.99	0.003
WC (cm)	85.48 ± 8.09	84.53 ± 7.93	87.38 ± 8.10	−2.79	0.006
SBP (mmHg)	120.03 ± 12.23	118.60 ± 11.91	122.87 ± 12.42	−2.77	0.006
DBP (mmHg)	76.66 ± 9.39	75.68 ± 9.12	78.59 ± 9.66	−2.44	0.015

FBS = fasting blood sugar; BMI = body mass index; WC = waist circumference; SBP = systolic blood pressure; DBP = diastolic blood pressure; t = independent t-test; χ^2^ = chi-square test

**Table 2:** Correlation of Health Behaviors and Occupational Stress.

Variables	Total (*N* = 275)	Normal (*N* = 183)	Prediabetic State (*N* = 92)
	*r* (*p*-Value)		
Health behaviors			
Smoking	0.22(<0.001)	0.16(0.032)	0.37(<0.001)
Alcohol consumption	0.30(<0.001)	0.21(0.004)	0.53(<0.001)
Physical activity	0.12(0.041)	−0.16(0.031)	0.34(0.001)
Diet	0.28(<0.001)	0.09(0.232)	0.58(<0.001)
Occupational stress			
Short form total	0.19(0.002)	0.11(0.136)	0.31(0.003)
Subscales			
Job demand	0.11(0.082)	0.09(0.218)	0.21(0.043)
Insufficient job control	0.08(0.217)	0.04(0.573)	0.05(0.641)
Interpersonal conflict	0.15(0.012)	−0.03(0.643)	0.25(0.017)
Job insecurity	0.13(0.031)	−0.05(0.512)	0.21(0.049)
Organizational system	0.28(<0.001)	−0.01(0.873)	0.27(0.010)
Lack of reward	0.06(0.301)	−0.06(0.402)	0.40(<0.001)
Occupational climate	0.11(0.072)	−0.08(0.288)	0.20(0.059)

**Table 3:** Factors Influencing Prediabetic State (*N* = 275).

**Variables**	**Model 1**			
	**B**	**SE**	**Wald F**	***p*-Value**
Health behaviors				
Alcohol consumption				
None or <1 drink	0.00			
1~4.9 drinks	0.17	0.30	0.30	0.584
≥5 drinks	1.65	0.70	5.57	0.018
Diet				
Both vegetables and fruits	0.00			
Only vegetables/only fruits	0.74	0.37	3.98	0.046
Neither vegetables nor fruits	0.93	0.38	6.00	0.014
Occupational stress				
Job insecurity				
1~2 Q	0.00			
3 Q	-	-	-	-
4 Q	0.53	0.29	3.39	0.065
Organizational system				
1~2 Q	0.00			
3 Q	1.01	0.39	6.67	0.010
4 Q	1.41	0.36	15.36	<0.001
**Variables**	**Model 2**			
	**B**	**SE**	**Wald F**	***p*-Value**
Health behaviors				
Diet				
Both vegetables and fruits	0.00			
Only vegetables/only fruits	0.77	0.39	3.97	0.046
Neither vegetables nor fruits	1.20	0.39	9.64	0.002
Occupational stress				
Short form total				
1~2 Q	0.00			
3 Q	0.15	0.42	1.16	0.048
4 Q	1.33	0.59	3.78	0.024
Organizational system				
1~2 Q	0.00			
3 Q	1.07	0.41	4.90	0.009
4 Q	1.13	0.43	6.84	0.009
Working years (years)	0.52	0.02	6.29	0.009
Waist circumference (cm)	0.47	0.19	6.37	0.012

Model 1 was nonadjusted and Model 2 was adjusted for working years, marital status, waist circumference, systolic blood pressure, and diastolic blood pressure. B = Unstandardized beta coefficient; SE = standard error; OR = odds ratio; CI = confidence interval; 1–2 Q = 1–2 Quartile; 3 Q = 3 Quartile; 4 Q = 4 Quartile.
